# Impact of umbilical cord mesenchymal stromal/stem cell secretome and cord blood serum in prostate cancer progression

**DOI:** 10.1007/s13577-023-00880-z

**Published:** 2023-02-19

**Authors:** André Sousa, Pedro Coelho, Fernanda Leite, Catarina Teixeira, Ana Catarina Rocha, Inês Santos, Pilar Baylina, Ruben Fernandes, Raquel Soares, Raquel Costa, Andreia Gomes

**Affiliations:** 1 Unit of Biochemistry, Department of Biomedicine, Faculty of Medicine, Universidade do Porto, Porto, Portugal; 2 i3S – Instituto de Investigação e Inovação em Saúde, Universidade do Porto, Porto, Portugal; 3 LaBMI – Laboratório de Biotecnologia Médica e Industrial, PORTIC – Porto Research, Technology, and Innovation Center, Polytechnic of Porto, Porto, Portugal; 4 School of Health (ESS), Polytechnic of Porto, Porto, Portugal; 5 Department of Clinical Haematology, Centro Hospitalar Universitário do Porto, Porto, Portugal; 6 FCS – Faculty of Health Sciences, HE-UFP, Hospital Escola – Universidade Fernando Pessoa, Porto, Portugal; 7 Bebé Vida, Ciências Para a Vida, S.A, Av. da França 476, 4050-367 Porto, Portugal

**Keywords:** Umbilical cord, Mesenchymal stem/stromal cells, Prostate cancer, Cord blood serum, Acellular therapy

## Abstract

Prostate cancer (PCa) is the second most common malignancy in men, and the fifth leading cause of death worldwide. Mesenchymal stromal/stem cells (MSC) have been identified in PCa, although contradictory effects in malignant transformation and tumor progression have been described. Since umbilical cord (UC) MSC and cord blood serum (CBS) are rich in numerous growth and anti-inflammatory factors, UC-MSC secretome and CBS are able to modulate tumor cell proliferation and survival as well as immunity and angiogenesis. In the present study, we address this relationship and investigate the influence of UC-MSC secretome and CBS on two human PCa cell lines (PC3 and LNCaP) and a normal epithelial prostate cell line (HPEpiC). Our results disclosed that upon exposure to UC-MSC-conditioned medium or CBS, both PC3 and LNCaP cells exhibited reduced viability, proliferation, and motility while non-malignant epithelial prostate cells were unaffected. These findings were corroborated by expression analysis of AKT/PI3K signaling pathway, p53 and interleukin genes. UC-MSC and CBS factors decreased the expression of growth-stimulating AKT and PI3K effectors and simultaneously up-regulated the expression of tumor-suppressor p53. Moreover, a more anti-inflammatory expression profile was found in both malignant PCa cell lines. Altogether, these results shed light into possible mechanisms by which UC-MSC and CBS reduce PCa progression, further reinforcing their potential use as novel therapeutic agents in PCa.

**Graphical abstract:**

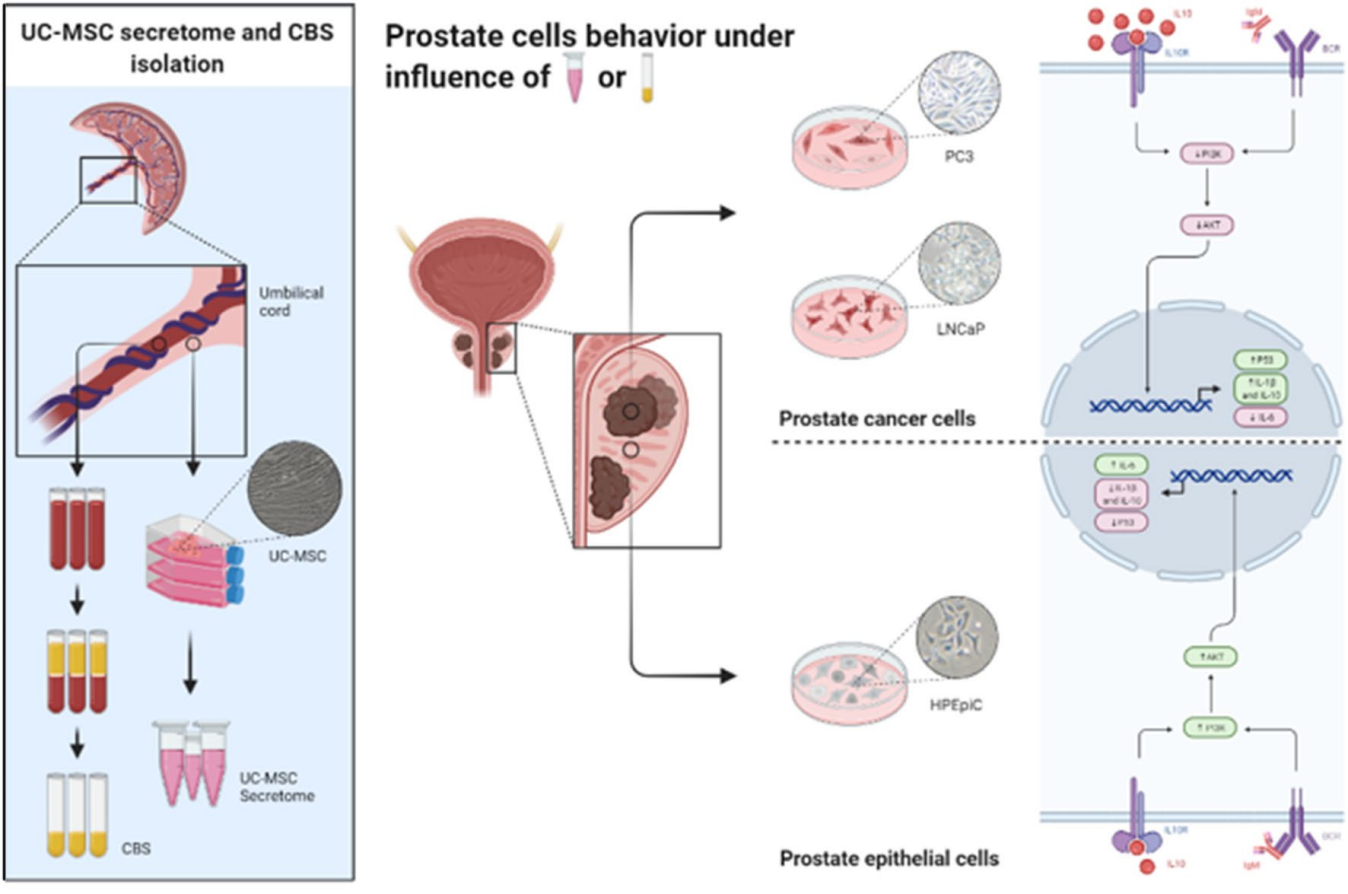

## Introduction

Prostate anatomical location potentiates several internal and external injuries, such as by infectious agents, carcinogens, urinary reflux, hormonal changes, and also physical trauma [1], that can progress to chronic inflammation, culminating in both initiation and progression of benign prostatic hyperplasia and prostate cancer (PCa) [1–3]. This carcinoma with indolent properties and with a non-invasive nature presents one of the highest rates of neoplastic transformation in the human body leading to death due not only to the anatomical disposition to damage, but also a failure in the control of metastatic process [1]. PCa affects more than 90% of men over 80 years and it is considered as a serious threat to the life of patients [4]. In fact, PCa is one of the five most prevalent cancers, being considered a significant health problem in both frequency and cancer-related death rate [5, 6]. Moreover, considering the aging and fast growth of world population, in 2030 it is expected that more than 1.7 million men will be diagnosed with PCa and around half a million new deaths by this condition [1]. The etiology of PCa is yet to be completely unraveled [7]. In addition, the biological heterogeneity of this cancer mirrors its interindividual, intertumoral, intratumoral, and genetical variations during treatment. Currently, there are a variety of treatments available against PCa such as surgery, androgen deprivation therapy, chemotherapy, radiotherapy, and active surveillance by prostate specific antigen. Nevertheless, the choice of the more suitable treatment combination is yet unclear, and the most common solution is the total prostatectomy [8]. The complexity of tumor microenvironment, radiation resistant stem cells, the increased secretion of inflammatory cytokines and growth factors, and overexpressed receptors impacts on cancer recurrence or on the appearance of resistant tumors [9]. Since the current therapies seem to have limited potential to prevent progression and treat PCa, the development of targeted, less toxic, and more efficient therapeutical strategies to control this disease is crucial, mainly for the metastatic potential.

The finding of unique properties of stem cells in modulating immunity, selective migration to inflammatory sites, and growth factors secretion, mesenchymal stem/stromal cells (MSC) have been considered a promising therapeutic avenue, namely in PCa [7, 10]. Indeed, stem cell therapy bring new paradigm for cancer inhibition by transporting chemotherapeutics, activating prodrugs, regulating the expression of genes involved in cancerigenesis, genetically modifying the production of anti-cancer agents, and deriving extracellular vesicles (EVs) containing therapeutic compounds from stem cells. Umbilical cord (UC) is currently considered an excellent and one of the earliest and most primitive source of human stem cells [7]. Therefore, both cord blood serum (CBS) and MSC isolated from the Umbilical Cord (UC-MSC) are derived from an easy-access, non-invasive, donor-risk free and non-controversial source that would normally be discarded [11, 12]. As reviewed by Gomes et al. (2021), UC-MSC are able to modulate several cellular mechanisms similarly to MSC cellular therapy, improving wound healing and tissue repair mechanisms in several disease models by promoting neovascularization [13]. In fact, UC-MSC secretome can be seen as an excellent therapeutic agent, avoiding the limitations of cellular approaches [10]. These acellular therapies are therefore based on the principle, already demonstrated, that the therapeutic effects associated with UC-MSC are mainly due to paracrine factors [14]. Which means that UC-MSC exert a large part of their biological effects through the release of biologically active molecules and EVs, being considered as an effective acellular alternative to UC-MSC [15]. In fact, the demonstrated therapeutic efficacy of MSCs is described to not depend on the physical proximity of the transplanted cells within tissues [16] but the synergistic action of small molecules secreted by MSCs has capacity of reducing cell injury and improving tissue repair [17, 18]. Therefore, besides direct cell differentiation and replacement therapy, UC-MSC secretome contains a strong paracrine component which can strongly be considered as a new paradigm towards cell-free therapeutics in medicine [19]. Furthermore, MSCs present the ability to migrate toward damaged tissues, to secrete bioactive mediators, such as growth factors, cytokines and EV that exert immunosuppressive, anti-apoptotic, anti-fibrotic, angiogenic, and anti-inflammatory effects [20]. Human MSCs have been showing benefits by improving ventricular function in a porcine model of myocardial ischemia [21]; by improving insulin sensitivity associated with an increased GLUT4 expression in type 2 diabetic rats [22]; through the prevention of neurocognitive impairments induced by cranial radiation in mice [23]; by reducing the T cell response leading to a beneficial effect in atopic dermatitis in a murine model [24]. These reports reveal two main properties of MSCs: the capacity to migrate toward the lesion site and the ability to repair damaged tissues.

The composition of UC-MSC secretome is variable and dependent on the microenvironment in which the UC-MSC are found, thus contributing to the multiplicity of effects reported in several studies and experimental conditions [14, 25]. Indeed, MSCs secrete cytokines being the most important IFNγ, TNFα, IL-1β and IL-6 [25]. Their effects depend on the local conditions of the microenvironment, and the pro-inflammatory IFNγ, TNFα and IL-1β cytokines may induce secretion of anti-inflammatory immunosuppressive molecules. The study of the expression of 120 cytokines at mRNA and protein levels of MSCs showed that IL-6 had the highest expression being the basic cytokine responsible for the immunoregulatory effects of MSCs [26]. IL-6 is a cytokine with a key role in a several cellular processes such as regulation of the immune response, hematopoiesis, inflammation, cell survival, apoptosis, cell proliferation and oncogenesis [25]. MSCs can transform malignant cells in an IL-6 dependent manner questioning the interactions between MSCs and the tumor microenvironment which is most commonly very rich in IL-6. Another MSC produced cytokine is transforming growth factor beta (TGFβ) with a key role in cellular functions, including proliferation, differentiation, migration, adhesion, and apoptosis, impacting important biological functions such as development, wound healing, carcinogenesis, angiogenesis, and immune responses [25]. TGFβ orchestrates the initiation and resolution of inflammatory responses, as well as the induction and maintenance of immune tolerance [25].

Although several studies have shown UC marked therapeutic potential in diverse pathologies, from hematology to immunology, and tissue regeneration, the role of some UC components in oncology field is not yet clear. Some reports have shown that the direct interaction between MSC and cancer cells potentiates metastatic capacity by enhancing epithelial–mesenchymal transition, a key event in tumor invasion process [27, 28]. Nevertheless, the available literature concerning this topic is contradictory [29]. For instance, some studies reported a pro-tumor effect of MSC by releasing growth or pro-angiogenic factors that potentiate the tumor growth contributing to cell progression [30, 31] and other works have demonstrated an anti-tumor effect by MSC by inhibiting proliferation-related signaling pathways, induction of cell cycle interruption and reduction of tumor growth [32, 33]. Despite the dual properties of UC-MSC in carcinogenicity and ability to inhibit tumor growth, CBS has been considered to have more potential in therapeutics than adult plasma since it contains higher levels of anti-inflammatory molecules [34], higher levels of soluble molecules responsible for NK, NKT, and T cells impairment [e.g. NK group 2, member D (NKG2D) and sNKG2DL] and responsible for directly downmodulated NK cell cytotoxicity in a dose-dependent manner [35]. CBS also exerts effects in proliferation, differentiation and function of immune and other cells types due to several growth factors, cytokines and immunomodulatory factors [36]. Moreover, this serum also contributes with a considerable panel of trophic factors (e.g. albumin, transferrin, fibronectin, several fatty acids and platelets) in wound healing mechanisms of epithelial cells [36]. Therefore, CBS therapeutic benefits associated to its availability through cord blood banking make this an ideal source for the treatment of a variety of diseases and inflammatory conditions [7].

Even though the effort to reach new treatment modalities which improve survival in PCa patients with metastasis, considering stem cell as therapy or even as co-adjuvant has not received much attention. Thus, we wanted to investigate the effects of both, CBS, and UC-MSC on cellular proliferation, viability, and function of human PCa. In this study, we evaluated the effect of both CBS and UC-MSC secretome on the behavior of androgen-sensitive human prostate adenocarcinoma (LNCaP) and androgen-nonresponsive grade IV prostate adenocarcinoma with high metastatic potential (PC3) cell lines. In parallel, the effect of both treatments was investigated on a non-malignant human prostate epithelial cell line (HPEpiC). Thus, the aim of the study was to evaluate the functionality and viability of each cell line by characterizing cytotoxicity, proliferation, and migration capacity under CBS and UC-MSC treatments, as well as the evaluation of a panel of survival and inflammatory genes.

## Methodology

### Sample collection

CBS and UC-MSC derived from UC units were obtained from BebeVida, Ciências para a Vida S.A. The informed consent for the use of samples for research was obtained in accordance with the requirements of the Declaration of Helsinki and local law at the time of collection.

CB and UC units were collected by qualified health care professionals in authorized maternity hospitals. Before delivery, mothers signed an informed consent donating these samples for both research and validation purposes.

Full serological and microbiological analysis of the CB units was carried out to exclude infection. Tests were performed for the presence of human immunodeficiency virus, *Treponema pallidum* (syphilis), cytomegalovirus and Hepatitis A, B, and C viruses, as well as aerobic and anaerobic bacterial and fungi (BacTAlert, Biomerieux, INC. Durham). Only non-reactive units were further processed.

### Cell culture

#### UC-MSC culture and characterization

UC-MSC were isolated from the umbilical cords of healthy neonates using the direct plastic adherence method. Briefly, the UC samples were sheared into long segments and washed to remove residual cord blood. Then, blood vessels and clots were scraped and washed away, and UC tissue was minced and digested using collagenase (Nordmark Biochemicals, German) and hyaluronidase (Sigma-Aldrich, Germany). Cells were then plated and incubated in a 5% CO2 atmosphere at 37 °C in MesenCult^™^ Basal Medium (STEMCELL Technologies, France) supplemented with 10% (v/v) MesenCult^™^ Stimulatory Supplement (STEMCELL Technologies, France) and 1% antibiotic-antifungal (v/v, Sigma-Aldrich, Germany). UC-MSC propagated to the 3rd–8th passages were characterized using accepted MSC-positive markers anti-CD73, CD90, and CD105 (BD Pharmingen, BD Biosciences, CA, USA), negative hematopoietic markers anti-CD45, CD34, CD11b, CD19, and HLA-DR (BD Stemflow PE hMSC Negative Cocktail, BD Biosciences, CA, USA) and cellular viability (BD 7-AAD, BD Biosciences, CA, USA) by flow cytometry (BD FACSCalibur™ Flow Cytometer; BD Biosciences, CA, USA) (data not shown).

#### Cell line culture

PCa cell lines PC3 and LNCaP were cultured in RPMI-1640 medium (VWR, Biowest, P0860-N10L, USA) supplemented with 10% of FBS (Gibco, Life technologies, 10,270, USA) and 1% penicillin/streptomycin (Gibco, Life technologies, 10,270, USA). HPEpiC cell line was cultured in Prostate Epithelial Cell Culture Medium (Innoprot, P60129, Spain) supplemented with 2% of FBS (Gibco, Life technologies, 10,270, USA), 1% of Prostate Epithelial Cell Growth Supplement (Innoprot, Spain) and 1% penicillin/streptomycin (Innoprot, Spain).

### Prostate cell lines treatment with UC-MSC secretome and CBS

Cord blood was transferred into the processing bag set which is placed in the AXP Device and then centrifuged. During centrifugation, component stratification and separation occurs. Red blood cells (RBC) are transferred to a separate sterile bag, the buffy coat which contains the mononuclear cell rich layer is delivered to a separate sterile freezing bag while the plasma remains in the processing bag [37]. Afterwards, the plasma was collected and centrifuged to remove all debris and stored at – 20 °C for subsequent treatments.

After primary culture, UC-MSC were propagated and allowed to reach confluence. Then, cultures were washed with HBSS (Hanks' Balanced Salt solution) and incubated in serum-free Dulbecco’s modified Eagle’s medium (DMEM). After 24 h, the conditioned medium was harvested from the UC-MSC cultures, spun for 5 min at 300 g and the supernatant was stored at – 20 °C for the subsequent treatments.

Treatments were performed for 24 h, using a final concentration of 10%, 20% and 25% of CBS or 25%, 50%, 75% and 100% of UC-MSC secretome. All treatments were performed in serum-free media and accomplished after a starvation period of 4 h.

### Cytotoxicity assay

PC3 (1 × 10^5^ cells/mL), LNCaP (2 × 10^5^ cells/mL) and HPEpiC (1 × 10^5^ cells/mL) were cultured and submitted to the different treatments. Then, MTT [3-(4,5-dimethylthiazol-2-yl)-2,5-diphenyl tetrazolium bromide] (Abcam, Cambridge, UK) was incubated at a final concentration of 0.5 mg/mL with incomplete cell culture medium for 3 h at 37 °C and 5% CO_2_. The absorbance was then measured using a microplate reader at 570 nm (Multiskan Ascent^®^, ThermoFisher Scientifics). Results were performed in triplicates and normalized to the control treatment (considered to be 100%).

### Proliferation assay

The BrdU kit from Roche Diagnostics was used to perform the colorimetric bromodeoxyuridine (BrdU) assay (Rotkreut, Switzerland), which evaluates the capacity of proliferating cells incorporate the BrdU. Briefly, PC3 (1 × 10^5^ cells/mL), LNCaP (2 × 10^5^ cells/mL) and HPEpiC (1 × 10^5^ cells/mL) were seeded and submitted to treatment period with 0.01 mM BrdU for 24 h. Optical density of proliferating cells was measured at 660 nm. Results were performed in triplicates and normalized as a percentage of the control treatment.

### Injury assay

Injury assay was used to evaluate cell migratory parameters such as speed and cell motility. For that, PC3, LNCaP and HPEpiC were seeded until confluence and then wounded with a pipette tip. After that, treatments were applied, and the injured cell monolayer was photographed at a magnification of 100 × with an inverted microscope (Nikon Instruments Inc., Melville, USA). The migrating distance was imaged 24 or 48 h after treatment, and the scratch closure was calculated by measuring the damage width with Image J software (U. S. National Institutes of Health, Bethesda, USA).

### mRNA extraction

Total RNA was extracted using NZYol^®^ (Life Technologies Invitrogen, USA), according to the manufacturer’s instructions. Briefly, PC3 (4 × 10^5^ cells/mL), LNCaP (8 × 10^5^ cells/mL) and HPEpiC (4 × 10^5^ cells/mL) were seeded, treated, and then subjected to extraction using NZYol. Total RNA was then separated from other organic components with chloroform, precipitated in isopropanol, washed with 75% ethanol, and sequentially solved in DEPC-treated H_2_O (Life Technologies Fishersci, USA). RNA purity was measured in nanodrop microplate spectrophotometer (Thermo Scientific^™^ Multiskan SkyHigh Microplate Spectrophotometer, Life Technologies Fishersci***, USA) by A260/A280 and A260/A230 ratio to verify RNA integrity.

### One-step RT-qPCR

Gene expression was estimated measuring the mRNA from cell extraction by real-time quantitative PCR (RT-qPCR) with qTOWER^3^ (Analytik Jena, GER), using One-step NZYSpeedy RT-qPCR Green kit (NZYTech, Portugal). Accordingly, 10 ng/µL RNA were employed, and the threshold cycle (TC) values from each biological assay were plotted with two experimental replicates for each biological sample, following the manufacturer's procedure. Melting curve analysis was used to monitor the specificity of primers, and results were normalized to GAPDH expression. Table 1 lists the primers sequences used.

**Table 1:** Set of primer sequences for the one-step multiplex RT-qPCR

Gene	Primer forward	Primer reverse
AKT1	TTCTGCAGCTATGCGCAATGTG	TGGCCAGCATACCATAGTGAGGTT
PI3K	GGTTGTCTGTCAATCGGTGACTGT	GAACTGCAGTGCACCTTTCAAGC
p53	CAGATCCGTGGGCGTGA	CATCCTTGAGTTCCAAGGCCTCATTC
IL-1β	ACCTAGCTGTCAACGTGTGG	TCAAAGCAATGTGCTGGTGC
IL-6	TGTGTGAAAGCAGCAAAGAGG	TTTTCACCAGGCAAGTCTCC
IL-10	TGAAAACAAGAGCAAGGCCG	ATAGAGTCGCCACCCTGATG
18 s	ACCGCAGCTAGGAATAATGGA	CCTCAGTTCCGAAAACCA

### Statistical analysis

One-way analysis of variance (ANOVA), followed by pairwise comparisons with Bonferroni’s *post-hoc* test was used to evaluate the effects of different concentrations of CBS or UC-MSC secretome in each cell line. For the comparison between two conditions in each treatment, an independent *t* Student’s test was performed. Normality distribution of data and the homogeneity of variance were assessed by the Shapiro–Wilk and the Levene’s tests, respectively. All data are expressed as a % of control and are presented as mean ± SD. Statistical significance was assumed when *p* < 0.05.

## Results

### Functional evaluation of prostate cell lines under UC-MSC secretome influence

**Fig. 1:**
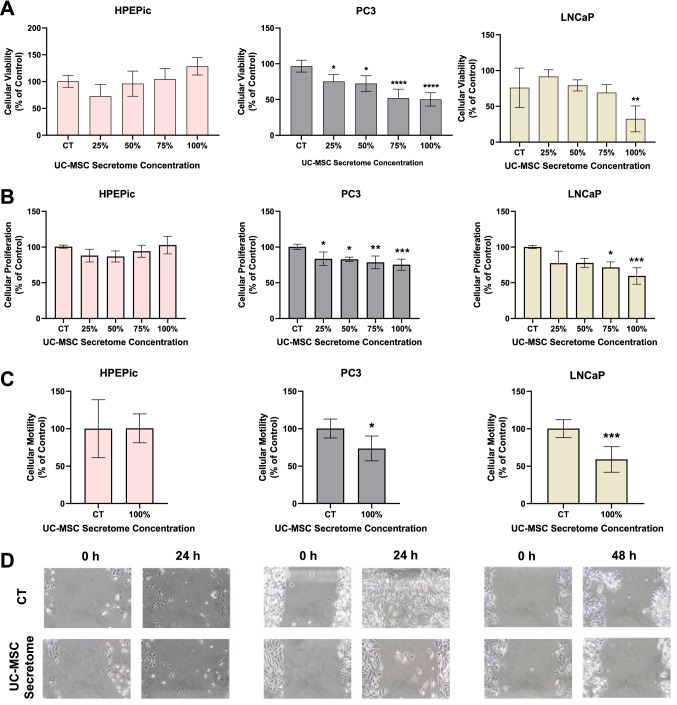
Functional assays under UC-MSC secretome. **A** Cytotoxicity rate in HPEpiC, PC3 and LNCaP cell lines and **B** proliferation rate in HPEpiC, PC3 and LNCaP cell lines for 0%, 25%, 50%, 75% and 100% UC-MSC secretome concentration. **C** Motility rate in HPEpiC, PC3 and LNCaP cell lines in the UC-MSC secretome concentration of 100%. **D** Migratory evaluation of HPEpiC, PC3 and LNCaP cell lines in the UC-MSC secretome concentration of 100%. Symbols above bars represent statistical significance (*p < 0.05; **p < 0.01; ***p < 0.001; ****p < 0.0001)

To evaluate the effect of UC-MSC secretome, cells were incubated with different concentrations. Cell viability, proliferation and migration were assessed after treatment in each cell line. Results are depicted in Fig. 1.

Concerning cell viability, depicted in Fig. 1A, in HPEpiC cell line no statistical differences were found between tested concentrations and control group. In PC3 cell line, UC-MSC decreases viability in a dose-dependent manner. In the concentration of 25%, a reduction of 24.9 ± 9.0% was observed and in the concentration of 50%, a reduction of 72.3 ± 10.1% was found (*p* < 0.05 *vs.* control group). Moreover, a reduction of cell viability was observed in the concentration of 75% and 100% in 52.0 ± 11.6% and 50.1 ± 8.6%, respectively (*p* < 0.0001 *vs.* control group). For LNCaP cell line, only the concentration of 100% of UC-MSC secretome led to a significant decrease of cell viability in 32.5 ± 15.6% (*p* < 0.01 *vs.* control group), with the other concentrations not reflecting a statistical difference compared to the control group.

Regarding cell proliferation represented in Fig. 1B, HPEpiC cell line shows no statistical differences between tested concentrations and control group once again. PC3 cell line shows a reduction of proliferation rate in all concentrations tested, noteworthy in the concentration of 25% and 50% in 16.4 ± 8.8% and 17.2 ± 2.8%, respectively (*p* < 0.05 vs. control group), in the concentrations of 75% in 21.3 ± 8.2% (*p* < 0.01 *vs.* control group) and in the concentration of 100% in 24.7 ± 7.2% (*p* < 0.001 *vs.* control group). In LNCaP cell line, the concentration of 75% leads to a reduction of 28.4 ± 6.8% (*p* < 0.05 *vs.* control group) and in the concentration of 100% decreases in 40.5 ± 10.5% (*p* < 0.001 *vs.* control group), with no statistical differences in the concentration of 25% and 50%.

Since the UC-MSC secretome concentration of 100% shows the higher reduction in viability and proliferation of tumoral cell lines, with no effect on normal prostate epithelial cell line, this concentration was employed to evaluate the effect in the migratory capacity of the three cell lines, depicted in Fig. 1C. In HPEpiC cell line, no statistical differences were found between the control group and the under UC-MSC secretome. In PC3 cell line, a reduction of 26.4 ± 16.5% was found in the presence of 100% secretome from stem cells (*p* < 0.05 *vs.* control group) and in LNCaP, a reduction of 40.9 ± 16.7% was observed (*p* < 0.001 *vs.* control group). Figure 1D represents the migratory process observed for the three cell lines, where control group of tumor cell lines migrates faster throughout the monolayer wound compared to the cells under UC-MSC secretome, contrary to what is observed for HPEpiC where cells from both experimental groups showed similar cell migration.

### Functional evaluation of prostate cell lines under CBS influence

**Fig. 2:**
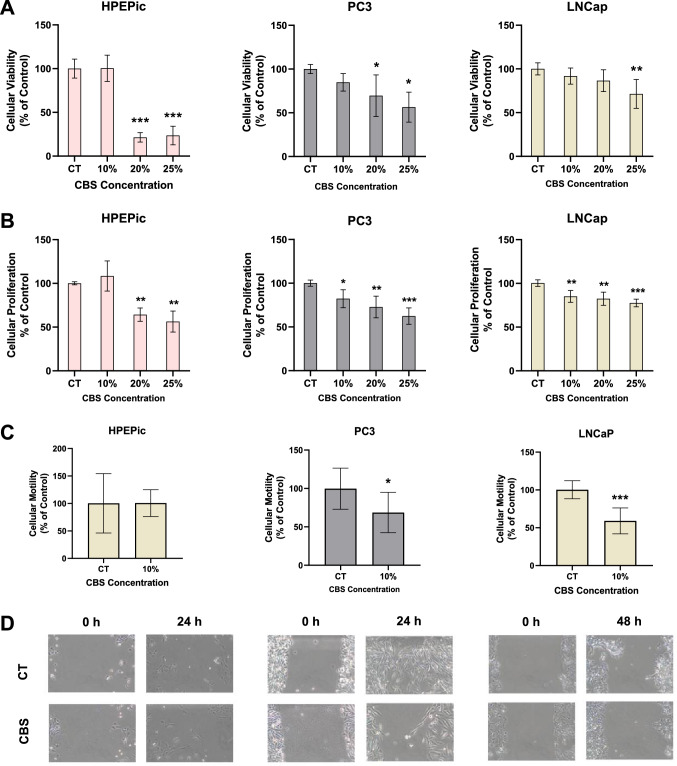
Functional assays under CBS concentrations. **A** Cytotoxicity rate in HPEpiC, PC3 and LNCaP cell lines. **B** Proliferation rate in HPEpiC, PC3 and LNCaP cell lines. **C** Motility rate in HPEpiC, PC3 and LNCaP cell lines in the CBS concentration of 10%. **D** Migratory evaluation of HPEpiC, PC3 and LNCaP cell lines in the CBS concentration of 10%. Symbols above bars represent statistical significance (*p < 0.05; **p < 0.01; ***p < 0.001)

After the treatment period with different concentrations of CBS, cell viability, proliferation and migration were assessed in each cell line to evaluate the effect of treatment. Results are depicted in Fig. 2.

Cell viability is depicted in Fig. 2A. In HPEpiC cell line, 20% and 25% shows a decrease of viability in 88.6 ± 5.0% and 86.4 ± 9.9%, respectively (*p* < 0.001 vs. control group), while the concentration of 10% shows no statistical difference. In PC3 cell line, CBS decreases viability in the concentration of 20% and 25% with a reduction of 30.5 ± 21.7% (*p* < 0.05 vs. control group) and 43.6 ± 15.4% (*p* < 0.01 *vs*. control group), respectively; moreover, no statistical differences were found in the CBS concentration of 10%. In LNCaP cell line, only the concentration of 25% decreased the cell viability with statistical significance in 28.6 ± 15.4% (*p* < 0.01 *vs.* control group).

In terms of cell proliferation shown in Fig. 2B, results followed the observed tendency of cell viability. In HPEpiC cell line, the higher concentrations of CBS, 20% and 25%, reduced the proliferation rate in 35.9 ± 7.1% and 43.7 ± 11.1%, respectively (*p* < 0.01 vs. control group). In PC3 cell line, every CBS concentration tested leaded to a reduction in cell proliferation: the 10% of CBS decreased cell proliferation in 17.7 ± 9.5% (*p* < 0.05 vs. control group), the 20% concentration of CBS reduced in 27.1 ± 11.5% (*p* < 0.01 *vs.* control group) and the concentration of 25% CBS diminished cell proliferation in 37.6 ± 8.7% (*p* < 0.001 *vs.* control group). Identically to PC3 cell line, the LNCaP cell line have presented a reduction of cell proliferation in tested concentrations of CBS: in the concentration of 10% and 20% the reduction of cell proliferation is 15.0 ± 6.3% and 17.6 ± 7.0%, respectively (*p* < 0.01 vs. control group), and in the concentration of 25%, cell proliferation is reduced in 22.4 ± 4.1% (*p* < 0.001 vs. control group).

Based in the fact that 10% of CBS did not have a statistical significance in the HPEpiC cell line viability and proliferation, and once this cell line is a normal prostate epithelial cell line, this concentration was chosen to evaluate the effect of CBS in the migratory capacity of the three cell lines, depicted in Fig. 2C. In HPEpiC cell line, no statistical significance was observed after the treatment period. On the other hand, in the tumor cell lines, statistical differences were found in PC3 cell line, a reduction of migratory capacity was observed, with a reduction in 31.3 ± 26.1% compared to the control group (*p* < 0.05 *vs.* control group); in the LNCaP cell line, the migratory capacity decrease in 40.9 3 ± 17.0% compared to the control group (*p* < 0.001 *vs.* control group). Figure 2D represents the migratory process observed for the three cell lines, where control group of tumor cell lines migrates faster throughout the monolayer wound compared to the cells under CBS. Contrary to what is observed for HPEpiC where cells from both experimental groups showed similar cell migration.

### Gene relative expression of signaling and inflammatory pathways

**Fig. 3:**
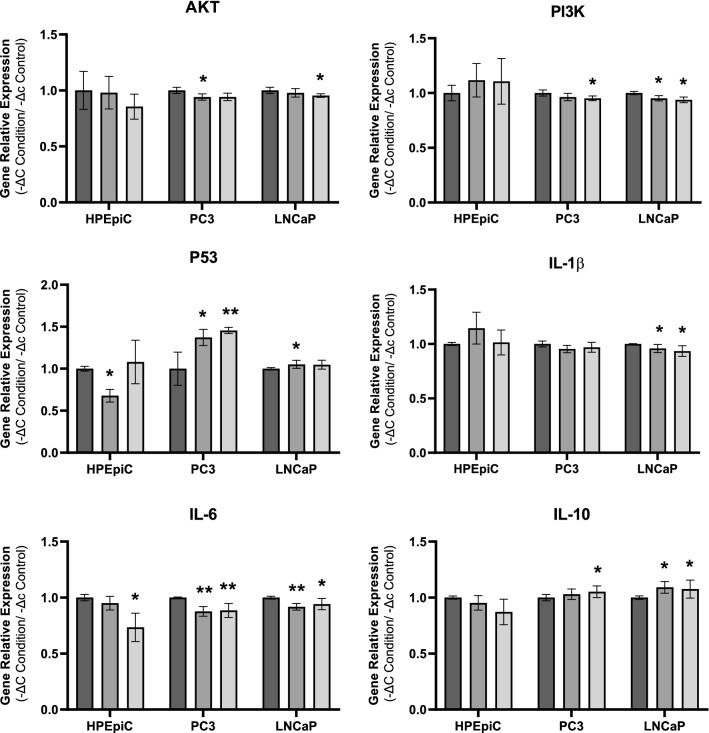
Gene relative quantification in HPEpiC, PC3 and LNCaP cell lines. **A** AKT. **B** PI3K. **C** p53. **D** IL-1β. **E** IL-6. **F** IL-10. Bars colors in (**A**)–(**E**) represents studied groups: groups:  Control group;  100% of UC-MSC;  10% of CBS. Symbols above bars represent statistical significance (*p < 0.05; **p < 0.01)

After treatment exposure, total RNA was extracted from each cell line and then used to relative quantification of signaling intermediates for cell proliferation, survival, and growth pathways (AKT, PI3K and p53), as well as to quantify pro and anti-inflammatory cytokines (IL-1β, IL-6 and IL-10). Results are depicted in Fig. 3.

Concerning AKT expression, although no statistical differences were found in HPEpiC cell line, PC3 shows a reduction of gene expression only in UC-MSC treatment in 5.9 ± 2.6% and LNCaP presents a reduction of gene expression in CBS treatment with a reduction of 4.6 ± 1.5% (*p* < 0.05 vs. control group). In terms of PI3K expression, no differences were found in HPEpiC cell line; in LNCaP cell line, a reduction of gene expression was found in UC-MSC treatment in 4.8 ± 2.2% and in both tumor cell lines, a reduction in gene expression was observed in CBS treatment in 4.8 ± 3.1% in PC3 cell line and 6.2 ± 2.3% in LNCaP cell line (*p* < 0.05 vs. control group). Regarding p53 expression, HPEpiC cell line shows a reduction of its expression under UC-MSC treatment in 32.2 ± 6.9% (*p* < 0.05 vs. control group) and no statistical differences were founded in CBS treatment; PC3 cell line presented an increase of this gene expression in both treatments, with an increase in 37.3 ± 9.0% in UC-MSC treatment (*p* < 0.05 vs. control group) and in 45.5 ± 3.5 in CBS treatment (*p* < 0.01 vs. control group); in LNCaP, an increase of gene expression was observed in UC-MSC treatment in 5.1 ± 4.4 (*p* < 0.05 vs. control group) and no statistical differences were found in CBS treatment.

Observing IL-1β expression, although no statistical differences were found in HPEpiC and PC3 cell line, LNCaP shows a reduction of gene expression in both treatments, with a decrease in 4.1 ± 3.4% in UC-MSC treatment and 6.5 ± 4.5% in CBS treatment (*p* < 0.05 vs. control group). Concerning IL-6 expression, in HPEpiC cell line no statistical differences were reported except upon the CBS treatment, where a reduction of 26.6 ± 11.6% was observed compared to the control group (*p* < 0.05 vs. control group); in PC3 cell line, both treatments reduced the expression of this gene, with a decrease in 12.3 ± 4.0% in UC-MSC treatment and in 11.4 ± 5.8% in CBS (*p* < 0.01 vs. control group); the same behavior was observed in LNCaP cell line, where both treatments decreased IL-6 expression in 8.2 ± 2.8% in UC-MSC (*p* < 0.01 vs. control group) and in 5.8 ± 4.7% in CBS (*p* < 0.05 vs. control group). Regarding IL-10 expression, no statistical differences were found in HPEpiC cell line; in LNCaP cell line, the UC-MSC treatment enhance this gene expression in 9.2 ± 4.9% and in both PCa cell lines (PC3 and LNCaP), CBS treatment led to an increase of this gene expression in 5.2 ± 4.8% for PC3 and in 7.7 ± 7.5% for LNCaP (*p* < 0.05 vs. control group).

## Discussion

The main findings of the present study are fourfold. First, UC-MSC secretome and CBS are able to significantly decrease cell viability, cell proliferation, inhibit the migratory behavior and decrease PI3K/AKT activation of both androgen-nonresponsive and androgen-sensitive human prostate adenocarcinoma cell lines. Second, the decrease of PI3K and AKT gene expression by UC-MSC secretome occurs differently in a cell-line dependent manner whereas CBS only impaired PI3K gene expression in PC3 PCa cells. Third, treatment of the PCa cell lines with UC-MSC secretome or CBS induced p53 wild type gene upregulation suggesting a regulatory effect on cell-cycle network. Finally, UC-MSC secretome decreased the expression of proinflammatory cytokines while increasing anti-inflammatory cytokines mRNA levels in cancer cells but not in normal human prostate epithelial cell line.

PCa impacts on the daily lives of men and imposes a high burden on health systems. Human UC-MSC are adult stem cells characterized by their low immunogenicity with high potential to be used for the development of novel therapeutic strategies as well as to be used as in vitro model [30]. Cumulative evidence has indicated that adult stem cells may be effective therapeutic tools for regenerative medicine and cancer therapy [30, 31].

Recent reports showed that UC-MSC have an intrinsic ability to attenuate the growth of several types of cancer cells [32–34]. Compared to bone marrow MSC, UC-MSCs have numerous advantages, including an improved expansion capacity, painless collection procedures, a lower risk of viral contamination and the fact that they are a possible source for autologous cell therapy [35]. Apart from direct cell differentiation and replacement therapy, UC-MSC release multiple bioactive proteins and compounds and secrete extracellular vesicles containing several regulatory factors [10]. Therefore, UC-MSC secretome and regulatory factors involve a strong paracrine component, leading to cell-free therapeutics in oncology.

This study uses techniques such the viability, proliferation and migration assessment to verify the reduction of tumoral features. The direct impact of the treatments in viability may be linked to cell death inducement or can be connected to the reduction in the proliferation capacity. Additionally, the evaluation of the migratory rate suggests the treatment potential to stimulate differences in cell motility, which are crucial for phenomena such as metastasis, characterizing PCa behavior. The results demonstrated that UC-MSC secretome and CBS are able to significantly decrease cell viability, cell proliferation, inhibit the migratory behavior and decrease PI3K/AKT activation of both cancer cell lines (LNCaP and PC3), even though 25% and 50% of UC-MSC secretome seemed to enhance the viability of the LNCaP cell line, which can derive from the secretome richness in metabolites and favorable factors (such as growth factors) that can activate proliferation pathways in this particular cell lines, overwhelming the anti-tumoral effects. Moreover, the neuroimmune network regulates tumor microenvironment and affect tumorigenesis, the molecules secreted by tumor microenvironment cells may activate signaling pathways with contradictory outcome such as tumor prone microenvironment [38]. Since the concentration of 100% UC-MSC secretome was the most promising to reduce tumor features without affecting the normal cell line, it was then hypothesized that UC-MSC secretome may interfere with the growth of tumor cells through regulating cell cycle arrest in different phases. These outcomes also suggest that the anticancer effect of UC-MSC secretome could depend on the specific cell types since UC-MSC secretome exerted marked effects in LNCaP when compared to PC3, which could be explained by the differences between both cell lines. PC3 is an androgen-nonresponsive grade IV prostate adenocarcinoma with high metastatic potential while LNCap is an androgen-sensitive human prostate adenocarcinoma cell line thus, more responsive to external stimuli than PC3 cells. These are promising findings because this treatment affected both PCa cell lines with no significant changes in normal epithelial cells (HPEpiC cell line). In fact, the proliferation enhancement tendency observed both with the use of CBS and UC-MSC secretome for HPEpiC cell line may be caused by the higher expression of the PI3K gene, which is directly involved in the proliferation capacity [39]. On the other hand, the higher concentrations of CBS led to higher cell death rates of HPEpiC cells. This effect can be the result of the accumulation of inflammatory factors or other molecules with the capacity to activate cell death pathways present in the CBS. In several cancers, PI3K/AKT pathway is overactive [39], thus reducing apoptosis and enhancing proliferation. In fact, aberrant PI3K/AKT pathway intermediates have putative roles in drug resistance development in PCa. Prostate tumors become resistant to androgen-deprivation therapy, unblocking the tumors ability to use the androgens to grow [39]. The results demonstrate that both UC-MSC secretome and CBS are able to significantly decrease PI3K/AKT activation in both LNCaP and PC3 cell lines, but in different ways. UC-MSC secretome decreases PI3K and AKT gene expression in LNCaP and PC3 cell lines, respectively. However, CBS was able to decrease both PI3K and AKT mRNA levels in LNCaP cell line but only significantly impaired PI3K gene expression in PC3. Furthermore, both UC-MSC secretome and CBS were able to increase p53 wild type gene expression, suggesting a marked effect on cell-cycle regulation. In fact, both UC-MSC and CBS factors were able to significantly decrease proliferation and increase apoptosis only in malignant prostate cells. Besides that, the fact that only UC-MSC secretome leads to a decrease in P53 expression in HPEpiC cell line can be connected to the fact that this secretome is reach in nutritive metabolites for the cells that inhibits the exacerbated activation of metabolic pathways that are regulated by the mentioned gene, such as lipogenic pathways, remaining with the use of basic metabolism such as glycolysis.

MSC are able to modulate the tumor microenvironment by several distinct mechanisms such as direct cell–cell interactions, or in a paracrine manner, through the secretion of cytokines and chemokines among stem cells and cancer cells [40, 41].

Considering the evaluation of the inflammatory cascades, the results showed that the UC-MSC secretome decreased the expression of proinflammatory cytokines while increasing anti-inflammatory cytokines mRNA levels. UC-MSC secretome did not affect the cytokine expression profile in HPEpiC cells and its effect was more noticeable in LNCaP than in PC3 cell line. PC3 cells are generally regarded as more aggressive than the LNCaP cell line, which can explain the differences in cytokine modulation by UC-MSC. These results corroborate the well-established UC-MSC effects towards inflammation. UC-MSC have the ability to modulate the immune system inhibiting immune cells proliferation, such as T cells and B cells; inducing macrophages switch from pro-inflammatory phenotypes to more anti-inflammatory phenotypes and reduce inflammation by secreting IL-10 and IL-4 [42]. UC-MSC also suppress the secretion of inflammatory factor IL-1β, TNF-α and IL-8, reducing inflammation and oxidative stress, thus suppressing cell apoptosis [43, 44]. CBS was also able to modulate cytokine expression profile. CBS has therapeutic advantages over adult rich-platelet plasma since it contains higher levels of anti-inflammatory factors. In fact, adult rich-platelet plasma contains more pro-inflammatory molecules which subsidize inflammation and aggravate pre-existent pathophysiological conditions [34]. Moreover, CBS contains several types of growth factors, cytokines and other immunomodulatory factors that can act on the proliferation, differentiation and function of immune and many other cells such as fibroblasts [45]. However, this study shows that CBS increased IL-10 gene expression in both cell lines and decreased IL-1β in LNCaP and IL-6 mRNA levels in both cell lines, promoting an anti-inflammatory profile similarly to UC-MSC secretome. Although protein expression levels were not evaluated in this study, strong correlations between mRNA and protein levels have been previously demonstrated by numerous studies for malignant cell lines [46, 47] [48]. In fact, the activation of the PI3K/AKT/mTOR signaling pathway showed similar pattern of expression either in mRNA or protein. Comparable correlations between mRNA expression and protein levels have also been described for IL-1β [49–52], Il-10 [53], and Il-6 [54]. Therefore, it is our conviction that the changes in mRNA levels found translate to a high extent to changes at the corresponding protein levels.

## Conclusion

Our results disclose that UC-MSC secretome and CBS reduce malignant prostate cell lines aggressiveness. The synergic effects on PCa survival, motility, and immunomodulation, allied to the cell-free nature of MSC secretome and CBS, support the beneficial effects of both CBS and UC-MSC-released factors. Moreover, UC-isolated MSCs have numerous advantages, when compared to those harvested from the bone marrow, including an improved expansion capacity, easier collection procedures, and lower risks of contamination, and they represent a possible source for autologous cell therapy, standing as a novel milestone in the development of active CB and UC-MSC secretome-derived anti-cancer biopharmaceutical formulations.

Further studies regarding the impact of the treatments on death signaling pathways of cancer cell lines, as well as the characterization of both UC-MSC secretome and CBS can potentially lead to the discovery of novel therapeutic methodologies to be used as non-cell-based cancer therapies.

## References

[1] Rawla P (2016). Epidemiology of prostate cancer. World J Oncol Elmer Press.

[2] Sharp VJ, Takacs EB, Powell CR (2010). Prostatitis: diagnosis and treatment. Am Fam Physician.

[3] Bachmann C (2015). Serenoa repens for benign prostatic hyperplasia. Schweiz Z für Ganzheitsmed/Swiss J Integr Med.

[4] Bott SN, KL, *Prostate Cancer*, E.P. Australia, Editor. 2015.

[5] Hulvat MC (2020). Cancer incidence and trends. Surg Clin North Am.

[6] Bray F (2018). Global cancer statistics 2018: GLOBOCAN estimates of incidence and mortality worldwide for 36 cancers in 185 countries. CA Cancer J Clin.

[7] Rahimi Tesiye M, Abrishami Kia Z, Rajabi-Maham H (2022). Mesenchymal stem cells and prostate cancer: a concise review of therapeutic potentials and biological aspects. Stem Cell Res.

[8] Litwin MS, Tan HJ (2017). The diagnosis and treatment of prostate cancer: a review. JAMA.

[9] Abbasian Ardakani A, Rajaee J, Khoei S (2017). Diagnosis of human prostate carcinoma cancer stem cells enriched from DU145 cell lines changes with microscopic texture analysis in radiation and hyperthermia treatment using run-length matrix. Int J Radiat Biol.

[10] Driscoll J, Patel T (2019). The mesenchymal stem cell secretome as an acellular regenerative therapy for liver disease. J Gastroenterol.

[11] Liau LL (2020). Characteristics and clinical applications of Wharton’s jelly-derived mesenchymal stromal cells. Curr Res Transl Med.

[12] Subramanian A (2015). Comparative characterization of cells from the various compartments of the human umbilical cord shows that the Wharton's jelly compartment provides the best source of clinically utilizable mesenchymal stem cells. PLoS ONE.

[13] Gomes A (2021). Human umbilical cord mesenchymal stem cells in type 2 diabetes mellitus: the emerging therapeutic approach. Cell Tissue Res.

[14] Shojaei S (2019). Effect of mesenchymal stem cells-derived exosomes on tumor microenvironment: tumor progression versus tumor suppression. J Cell Physiol.

[15] Sandonà M (2021). Mesenchymal stromal cells and their secretome: new therapeutic perspectives for skeletal muscle regeneration. Front Bioeng Biotechnol.

[16] Noiseux N (2006). Mesenchymal stem cells overexpressing Akt dramatically repair infarcted myocardium and improve cardiac function despite infrequent cellular fusion or differentiation. Mol Ther.

[17] Caplan AI, Dennis JE (2006). Mesenchymal stem cells as trophic mediators. J Cell Biochem.

[18] Yeo RWY (2013). Exosome: a novel and safer therapeutic refinement of mesenchymal stem cell. J Circulating Biomark.

[19] Bian D (2022). The application of mesenchymal stromal cells (MSCs) and their derivative exosome in skin wound healing: a comprehensive review. Stem Cell Res Ther.

[20] Salgado AJ (2010). Adipose tissue derived stem cells secretome: soluble factors and their roles in regenerative medicine. Curr Stem Cell Res Ther.

[21] Liu CB (2016). Human umbilical cord-derived mesenchymal stromal cells improve left ventricular function, perfusion, and remodeling in a porcine model of chronic myocardial ischemia. Stem Cells Transl Med.

[22] Si Y (2012). Infusion of mesenchymal stem cells ameliorates hyperglycemia in type 2 diabetic rats: identification of a novel role in improving insulin sensitivity. Diabetes.

[23] Soria B (2019). Human mesenchymal stem cells prevent neurological complications of radiotherapy. Front Cell Neurosci.

[24] Song JY (2019). Umbilical cord-derived mesenchymal stem cell extracts ameliorate atopic dermatitis in mice by reducing the T cell responses. Sci Rep.

[25] Eleuteri S, Fierabracci A (2019). Insights into the secretome of mesenchymal stem cells and its potential applications. Int J Mol Sci.

[26] Park CW (2009). Cytokine secretion profiling of human mesenchymal stem cells by antibody array. Int J Stem Cells.

[27] Zhang LN (2019). Fusion with mesenchymal stem cells differentially affects tumorigenic and metastatic abilities of lung cancer cells. J Cell Physiol.

[28] Melzer C, von der Ohe J, Hass R (2019). In vivo cell fusion between mesenchymal stroma/stem-like cells and breast cancer cells. Cancers.

[29] Hmadcha A (2020). Therapeutic potential of mesenchymal stem cells for cancer therapy. Front Bioeng Biotechnol.

[30] Lu L (2019). Bone marrow mesenchymal stem cells suppress growth and promote the apoptosis of glioma U251 cells through downregulation of the PI3K/AKT signaling pathway. Biomed Pharmacother.

[31] Ho IA (2013). Human bone marrow-derived mesenchymal stem cells suppress human glioma growth through inhibition of angiogenesis. Stem Cells.

[32] Lee C (2012). Exosomes mediate the cytoprotective action of mesenchymal stromal cells on hypoxia-induced pulmonary hypertension. Circulation.

[33] Pakravan K (2017). MicroRNA-100 shuttled by mesenchymal stem cell-derived exosomes suppresses in vitro angiogenesis through modulating the mTOR/HIF-1α/VEGF signaling axis in breast cancer cells. Cell Oncol (Dordr).

[34] Belderbos ME (2013). Plasma-mediated immune suppression: a neonatal perspective. Pediatr Allergy Immunol.

[35] Cox ST (2018). Functional characterisation and analysis of the soluble NKG2D ligand repertoire detected in umbilical cord blood plasma. Front Immunol.

[36] Samarkanova D (2020). Cord blood platelet rich plasma derivatives for clinical applications in non-transfusion medicine. Front Immunol.

[37] Solves P (2013). Qualitative and quantitative cell recovery in umbilical cord blood processed by two automated devices in routine cord blood banking: a comparative study. Blood Transfus.

[38] Cervantes-Villagrana RD (2020). Tumor-induced neurogenesis and immune evasion as targets of innovative anti-cancer therapies. Signal Transduct Target Ther.

[39] Park S (2018). PI3K pathway in prostate cancer: all resistant roads lead to PI3K. Biochim Biophys Acta Rev Cancer.

[40] Whiteside TL (2018). Exosome and mesenchymal stem cell cross-talk in the tumor microenvironment. Semin Immunol.

[41] Hass R (2020). Role of MSC in the tumor microenvironment. Cancers.

[42] Dabrowski FA (2017). Comparison of the paracrine activity of mesenchymal stem cells derived from human umbilical cord, amniotic membrane and adipose tissue. J Obstet Gynaecol Res.

[43] Sun X (2017). Human umbilical cord-derived mesenchymal stem cells ameliorate insulin resistance by suppressing NLRP3 inflammasome-mediated inflammation in type 2 diabetes rats. Stem Cell Res Ther.

[44] Yao J (2019). Extracellular vesicles derived from human umbilical cord mesenchymal stem cells alleviate rat hepatic ischemia-reperfusion injury by suppressing oxidative stress and neutrophil inflammatory response. Faseb j.

[45] Versura P (2013). Efficacy of standardized and quality-controlled cord blood serum eye drop therapy in the healing of severe corneal epithelial damage in dry eye. Cornea.

[46] Riquelme I (2016). The gene expression status of the PI3K/AKT/mTOR pathway in gastric cancer tissues and cell lines. Pathol Oncol Res.

[47] Zhou J (2012). Genetic and bioinformatic analyses of the expression and function of PI3K regulatory subunit PIK3R3 in an Asian patient gastric cancer library. BMC Med Genom.

[48] Creighton CJ (2010). Proteomic and transcriptomic profiling reveals a link between the PI3K pathway and lower estrogen-receptor (ER) levels and activity in ER+ breast cancer. Breast Cancer Res.

[49] Dasu MR, Devaraj S, Jialal I (2007). High glucose induces IL-1beta expression in human monocytes: mechanistic insights. Am J Physiol Endocrinol Metab.

[50] Chen MF (2012). Role of interleukin 1 beta in esophageal squamous cell carcinoma. J Mol Med (Berl).

[51] Kai H (2005). Involvement of proinflammatory cytokines IL-1beta and IL-6 in progression of human gastric carcinoma. Anticancer Res.

[52] Deans DA (2006). Elevated tumour interleukin-1beta is associated with systemic inflammation: a marker of reduced survival in gastro-oesophageal cancer. Br J Cancer.

[53] Chen L (2019). IL-10 secreted by cancer-associated macrophages regulates proliferation and invasion in gastric cancer cells via c-Met/STAT3 signaling. Oncol Rep.

[54] Jiang XP (2011). Down-regulation of expression of interleukin-6 and its receptor results in growth inhibition of MCF-7 breast cancer cells. Anticancer Res.

